# The impact of Twitter/X promotion on visibility of research articles: Results of the #TweetTheJournal study

**DOI:** 10.1016/j.ijcha.2023.101328

**Published:** 2023-12-23

**Authors:** Konstanze Betz, Melania Giordano, Henrike Aenne Katrin Hillmann, David Duncker, Dobromir Dobrev, Dominik Linz

**Affiliations:** a Department of Cardiology, Maastricht University Medical Centre and Cardiovascular Research Institute Maastricht, Maastricht, the Netherlands; b Department of Internal Medicine, Eifelklinik St. Brigida GmBH & Co KG, Simmerath, Germany; c IJC Milan Srls, Meina, Novara, Italy; d Hannover Heart Rhythm Center, Department of Cardiology and Angiology, Hannover Medical School, Hannover, Germany; e Institute of Pharmacology, West German Heart and Vascular Center, University Duisburg-Essen, Germany; f Montréal Heart Institute, University de Montréal, Montréal, Quebec, Canada; g Department of Integrative Physiology, Baylor College of Medicine, Houston, USA; h Department of Biomedical Sciences, Faculty of Health and Medical Sciences, University of Copenhagen, Copenhagen, Denmark; i Netherlands Heart Institute, Utrecht, Netherlands

**Keywords:** Cardiovascular articles, Social media, Altmetrics, Mendeley reader, Randomized study, Citations

## Abstract

**Aim:**

Social media (SoMe) are emerging as important tools for research dissemination. Twitter/X promotion has been shown to increase citation rates in well-established journals. We aimed to test the effect of a SoMe promotion strategy on the Mendeley reader counts, the Altmetric Attention Score and the number of citations in an upcoming open-access journal.

**Methods:**

The #TweetTheJournal study is a randomized, controlled study. Articles published in seven subsequent issues of the International Journal of Cardiology Heart & Vasculature (April 2021-April 2022) were randomized to a Twitter/X promotion arm (articles were posted four times) and to a control arm (without active posting). Articles with accompanied editorials were excluded. Primary endpoint of the study was Mendeley reader count, secondary endpoints were Altmetric Attention Score and number of citations. Follow-up was one year.

**Results:**

SoMe promotion of articles showed no statistically significant difference in Mendeley reader counts or number of citations at one year follow up. SoMe promotion resulted in a statistically significant higher Altmetric Attention Score in the intervention compared to the control group (RR 1.604, 95 % CI 1.024–2.511, p = 0.039). In the overall group, Altmetric Attention Score showed a correlation with Mendeley reader counts (Spearman’s ρ = 0.202, p = 0.010) and Mendeley reader counts correlated significantly with number of citations (Spearman’s ρ = 0.372, p < 0.001).

**Conclusion:**

A dedicated SoMe promotion strategy did not result in statistically significant differences in early impact indicators as the Mendeley reader count in a upcoming journal, but increased the Altmetric Attention Score.

## 1 Introduction

Social media is evolving as an important component of modern science – including e.g. research dissemination, medical education and discussion around cardiovascular research [1]. Amongst a variety of tools available, the short-message platform Twitter (now “X”) emerged as the most popular for knowledge dissemination among cardiologists and researchers [2], [3], [4], [5], [6].

Hence, metrics such as the Altmetric Attention Score have been developed to measure the online-impact of scientific papers, and Twitter/X has been shown to have a high impact on the Altmetric Attention Score [7]. A social media promotion strategy, mostly through Twitter/X, is widely used by cardiovascular journals. Even the translation of Twitter/X based education into formal CME credits is currently discussed [4], [8].

A recently published randomized trial addressing the official journals of the European Society of Cardiology (ESC) showed that a dedicated Twitter/X promotion resulted in a higher Altmetric Attention Score and a higher number of citations [9]. Although the study showed a significant impact of Twitter/X promotion on number of citations, this positive effect on classical impact measures as the number of citations is not a consistent finding [10], [11], [12] and it remains unclear whether it also applies to open-access articles and to journals with lower intrinsic citation potential.

The International Journal of Cardiology Heart & Vasculature (IJC H&V) is an upcoming online-only, open access journal, where a Twitter/X promotion strategy was implemented in 2019. In 2021 (April 2021 – April 2022) the randomized #TweetTheJournal study was initiated to investigate the impact of a Twitter/X promotion strategy on early indicators of long-term impact of original manuscripts published in this journal, which was first listed for journal impact factor in 2022 [13].

## 2 Methods

### 2.1 Trial design

The #TweetTheJournal study was a randomized controlled trial. Original articles from subsequent issues were randomized per issue into a Twitter/X promotion arm (intervention group) and a control group. Articles were excluded if an editorial was accompanying them, otherwise no selection of articles was performed. Randomization was performed by using Sealed Envelope (https://www.sealedenvelope.com/).

The intervention included an active Twitter/X promotion of the original article through the official IJC H&V Twitter/X handle (@IJC_Heart_Vasc). The tweeting schedule was based on strategies used in previous studies [9], [10], [11] and has been published elsewhere [13]. Articles within the intervention arm were tweeted after publication of the issue and retweeted three different times until release of the subsequent issue (within 8 weeks) by the official Twitter/X handle of IJC H&V. Tweets were shared by official Twitter/X accounts of co-authors. To increase visibility and avoid possible bias, tweets and retweets by the official journal Twitter/X handle were posted at different daytimes and weekdays. For example, tweets were sent once in the morning, once at noon and once in the evening at three different weekdays (Example: tweet one Monday 8 am, tweet number two on Wednesday at 5 pm). Weekends (beginning Friday) and international holidays were excluded. To maintain consistency, layouts of the tweets were designed as uniformly as possible. Tweets were written in English including a short title, 4–5 hashtags referring to the article topic (including the hashtag “ICYMI” – in case you missed it) and a short link to the open-access article. Matching hashtags were derived from the Healthcare Hashtag Project Cardiology Ontology (https://www.symplur.com/healthcare-hashtags/ontology/cardiology/). Short links to the open-access articles were created by using Bitly (https://www.bitly.com). Further, each tweet included a visual abstract or - in case of absence of a visual abstract - a central figure taken from the original manuscript in high resolution quality. The Elsevier Journal Twitter/X handles @ELS_Cardiology and @ElsevierNews were tagged, as well as the official Twitter/X accounts of co-authors, if available. Within the control group, no social media promotion through the official journal Twitter/X handle was performed. Primary endpoint of the study was Mendeley reader count, which is the number of Mendeley users who added copies of a manuscript to their personal Mendeley library, a reference management software. Whereas citations are considered as indicators for long-term impact, the number of Mendeley readers appears earlier than citations and is therefore considered as early indicator of long-term impact of a scientific manuscript [13]. Secondary endpoints included number of citations and Altmetric Attention Score. Follow-up time was one year after.

### 2.2 Data collection

Mendeley reader count, number of citations and Altmetric Attention Score were collected at two time points (September 2022 for manuscripts published until end September 2021 and April 2023 for articles published until end of April 2022). Data were obtained from the online analytic tools PlumX Metrics (https://plumanalytics.com/learn/about-artifacts/) and Dimensions (https://app.dimensions.ai/discover/publication).

### 2.3 Statistics

Kolmogorov Smirnov test and Shapiro Wilk test were performed to assess normal distribution of data. Continuous variables did not underlie a normal distribution and therefore data are represented as median and interquartile range (IQR) in descriptive statistics additionally to count (n) and percentages (%) for categorical variables. Chi-Square Test and Fishers exact test were used for comparison of nominal, Mann Whitney *U* test for continuous data. Moreover, Spearman correlation was assessed. Assumption of equal variances was assessed using Levenes test. Since data was overdispersed, negative binomial regression analysis was performed. To account for differences in length of observation time, time (in days) since online publication was included as offset variable (logarithmic). Results were reported with incidence rate ration (RR) and 95 % Confidence Interval (CI). A p-value of 0.05 was considered statistically significant. For database management and statistical analysis IBM SPSS Statistics Version 27 was used.

## 3 Results

**Fig. 1:**
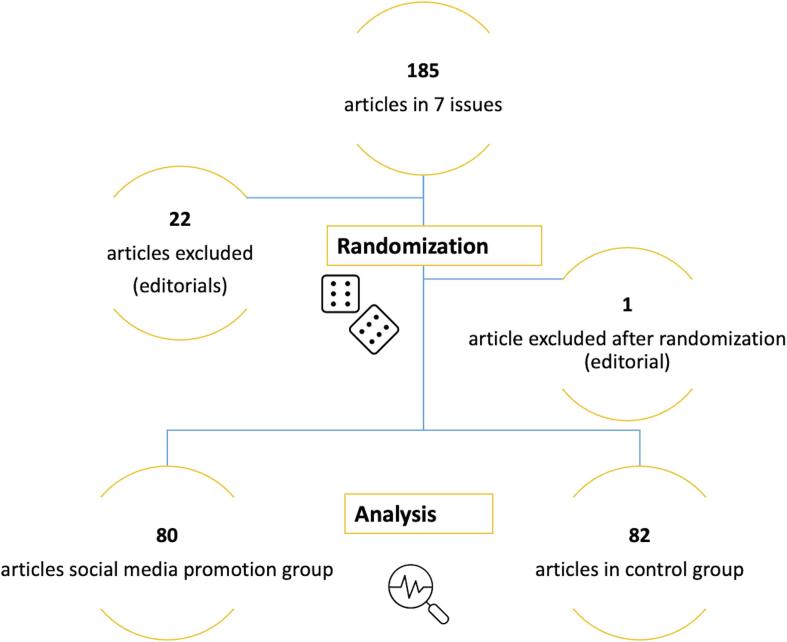
Study flowchart.

In the herein presented analysis, seven subsequent issues of the IJC H&V with a total of 185 articles were suitable for randomization. A total of 23 articles (12.4 %) were excluded, because in these articles, an editorial was accompanying them in the same or a following issue. Overall, 80 articles (43.2 %) were randomized to the Twitter/X and 82 articles (44.3 %) to the control group (Fig. 1). Overall, the majority of articles (81.5 %) were original manuscripts for clinical research and were not related to Covid-19 (94.4 %). The major part of first-author affilations were from countries in Europe and the United States (48.8 %). Median days between online publication and retrieval of data was 467 (1 0 5) days. Median number of tweets (including retweets) was 9 (12) and median upper bound of followers (potential reach on Twitter/X) was 5,457 (17,003). In the intervention and control group, there were no statistically significant differences between the characteristics of the articles in terms of type of research, whether they were Covid-19 related, corresponding author affiliations (Europe and United States vs Other) and days between online publication and retrieval of data (Table 1).

**Table 1:** Characteristics of papers in intervention (Twitter/X) *versus* control group (No-Twitter/X).

	*Overall (n = 162)*	Twitter/X (n = 80)	Control (n = 82)	p-value
	*n (%)*	n (%)	n (%)	
**Type of research**				0.483
Basic	*17 (10.5 %)*	8 (10.0 %)	9 (11.0 %)	
Clinical	*132 (81.5 %)*	64 (80.0 %)	68 (82.9 %)	
Review	*10 (6.2 %)*	5 (6.3 %)	5 (6.1 %)	
Other	*3 (1.9 %)*	3 (3.8 %)	0 (0.0 %)	
**COVID-19 related**				0.496
No	*153 (94.4 %)*	77 (96.3 %)	76 (92.7 %)	
Yes	*9 (5.6 %)*	3 (3.8 %)	6 (7.3 %)	
**Country of corresponding author**				0.608
Europe	53 (32.7%)	25 (31.3%)	28 (34.1%)	
United States	30 (18.5%)	13 (16.3%)	17 (20.7%)	
Other	79 (48.8%)	42 (52.5%)	37 (45.1%)	
**Days between release of issue and retrieval of data (median (IQR))**	*467 (1 0 5)*	475 (1 0 6)	457 (1 0 4)	0.455
**Number oftweets (total) (median (IQR))**	*9 (12)*	12 (5)	1 (4)	<0.001
**Upper bound of Twitter/X followers (median (IQR))**	*5457 (17003)*	11,266 (29524)	424 (7614)	<0.001

### 3.1 Effects of Twitter/X promotion on early impact indicators

**Fig. 2:**
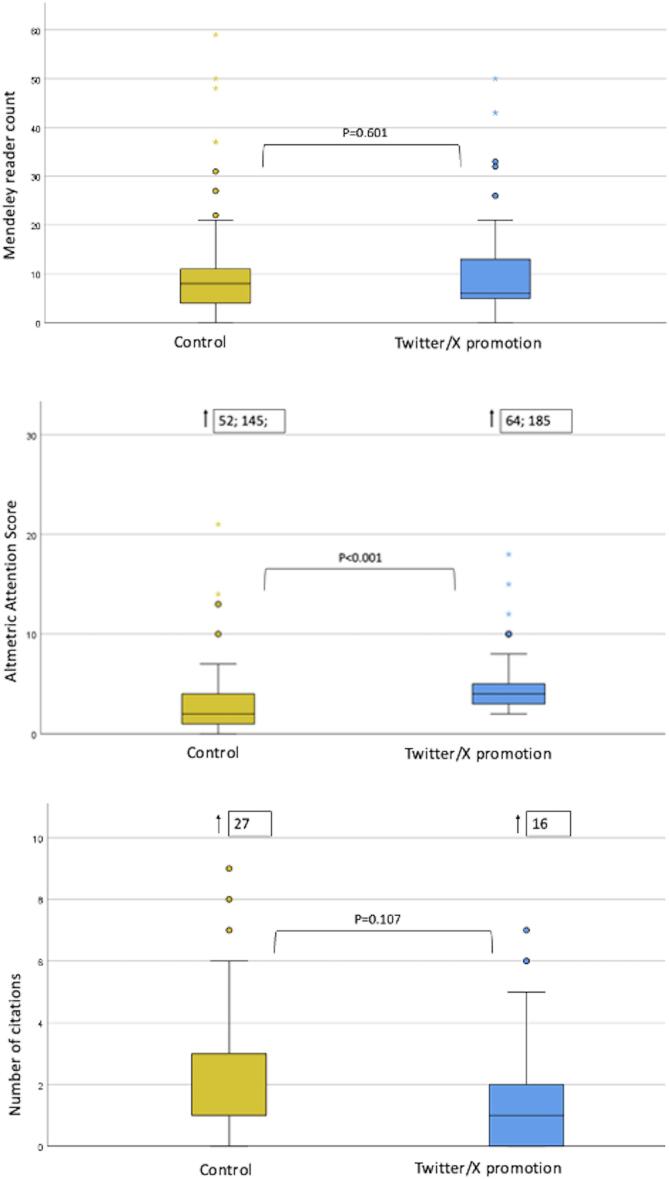
**Boxplot of Mendeley reader count, Altmetric Attention Score, number of citations in control *versus* social-media promotion group.** Boxplots are rescaled and textboxes next to arrows show absolute values outside the scaled Y-achsis.

The overall Mendeley reader count after approximately one year was 7 (IQR 8) with no statistically significant differences between both groups (6 (IQR 8) in the Twitter/X group and 8 (IQR 7) in the control group, p = 0.601 (see Fig. 2 and Table 2), with RR 1.049 (95 % CI 0.783–1.406; p = 0.750) of Twitter/X vs. control Mendeley reader count in negative binomial regression.

**Table 2:** Comparison of Mendeley reader count (primary endpoint) and secondary endpoints.

	*Overall (n = 162)*	Twitter/X (n = 80)	Control (n = 82)	p-value
**Mendeley reader count (median (IQR))**	*7 (8)*	6 (8)	8 (7)	0.601
**Altmetric Attention Score** **(median (IQR))**	*3 (3)*	4 (2)	1 (3)	<0.001
**Number of citations (median (IQR))**	*1 (2)*	1 (2)	1 (2)	0.107

The overall median Altmetric Attention Score was 3 (IQR 3) – 4 (IQR 2) in the Twitter/X group and 1 (IQR 3) in the control group, with a statistically significant difference between both groups (p < 0.001, see Fig. 2 and Table 2). Negative binomial regression analysis showed a RR 1.604 (95 % CI 1.024–2.511, p = 0.039) for Twitter/X vs. control for the Altmetric Attention Score.

The overall number of citations after one year was 1 (IQR 2) with no statistically significant differences between intervention and control group (median citation 1 (IQR 2) in Twitter/X and control group, respectively, p = 0.107, see Fig. 2 and Table 2). Negative binomial regression analysis showed a RR 0.742 (95 % CI 0.517–1.064; p = 0.105) for Twitter/X vs. control for number of citations.

### 3.2 Correlations

**Fig. 3:**
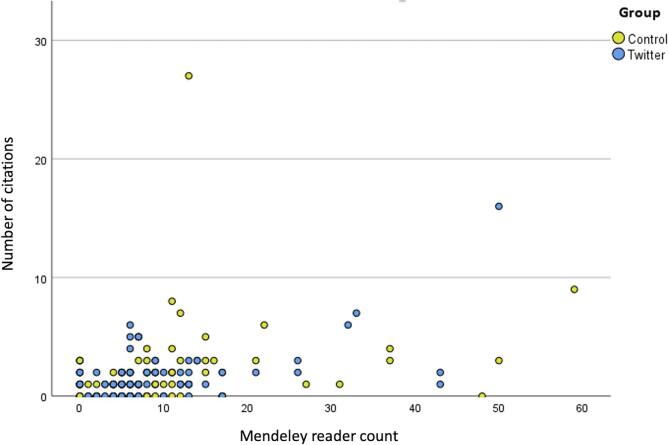
Scatterplot Mendeley reader counts and number of citations in overall group and both groups. Overall: Spearman’s ρ = 0.372, p < 0.001, social-media promotion group: Spearman’s ρ = 0.400, p < 0.001 Control group: Spearman’s ρ 0.392, p < 0.001 (no statistically significant difference in correlation coefficients between both groups).

In the overall group, Spearman correlation revealed statistically significant correlations between Mendeley reader counts and Altmetric Attention Score (Spearman’s ρ = 0.202, p = 0.010) and Mendeley reader count and number of citations (Spearman’s ρ = 0.372, p < 0.001, see Fig. 3) Moreover, number of citations correlated positively with time since publication (Spearman’s ρ = 0.192, p = 0.014) (Table 3a).

**Table 3a:** Correlation table for the overall group.

	Mendeley reader count	Number of citations	Altmetric Attention Score	Time since online publication
Mendeley reader count				
Number of citations	0.372^**^			
Altmetric Attention Score	0.202*	0.029		
Time since online publication	0.137	0.192*	0.026	

N = 162, *p < 0.05, **p < 0.001.

Within the Twitter/X and control groups, statistically significant correlation between Mendeley reader count and number of citations was observed in both groups, with stronger correlation in the intervention group (Spearman’s ρ = 0.400, p < 0.001, vs Spearman’s ρ 0.392, p < 0.001, see Fig. 3 and Table 3b). However, the correlation coefficients did not differ significantly between both groups (Z_OBS_ = -0.06).

**Table3b:** Correlation table divided in subgroups (Twitter/X and control).

		Mendeley reader count	Number of citations	Altmetric Attention Score	Time since online publication
**Control** N = 82	Mendeley reader count				
	Number of citations	0.392^**^			
	Altmetric Attention score	0.243*	0.133		
	Time since online publication	0.231*	0.323^**^	0.119	
**Twitter/X** N = 80	Mendeley reader count				
	Number of citations	0.400^**^			
	Altmetric Attention Score	*0.216^#^*	0.137		
	Time since online publication	0.012	0.079	−0.163	

*p < 0.05, **p < 0.001, ^#^ p = 0.055; no statistically significant differences in correlation coefficients were observed between both groups.

There was a statistically significant correlation between Mendeley reader counts and Altmetric Attention Score in the control group (Spearman’s ρ = 0.243, p = 0.028, see Table 3b) and a borderline significance in the intervention group (Spearman’s ρ = 0.216, p = 0.055, see Table 3b), but no statistically significant difference between correlation coefficients (Mendeley reader count/Altmetric Attention Score: Z_OBS_ = 0.177). In the control group, the correlation between time since publication and number of citations (Spearman’s ρ = 0.323, p = 0.003, see Table 3b) and correlation between time since publication and Mendeley reader count (Spearman’s ρ = 0.231, p = 0.036, see Table 3b) were statistically significant. The latter was not observed in the intervention group (time since publication/number of citations: Spearman’s ρ = 0.079, p = 0.486; time since publication/Mendeley reader counts: Spearman’s ρ = 0.012, p = 0.917, see Table 3b). Correlation coefficients were similar in both groups (time from publication/number of citations: Z_OBS_ = 1.62, time from publication/Mendeley reader counts: Z_OBS_ = 1.41).

### 3.3 Qualitative analysis of top-performing articles

For a qualitative analysis, all articles, including those with editorials, from issues 33–39 of 2021–2022 (n = 186) were analysed focusing on their performance in terms of Mendeley reader count, Altmetric Attention Score and number of citations. In the article with the highest Altmetric Attention Score, Pendell Meyers et al. describe the accuracy of OMI ECG findings *versus* STEMI criteria for diagnosis of acute coronary occlusion myocardial infarction [14]. This article was further in the top 3 articles in all three categories. The highest number of Mendeley readers was reached by an article of Zhao et al., which described the virtual multidisciplinary care for heart failure patients with cardiac resynchronization therapy devices during the Covid-19 pandemic [15]. The highest number of citations at follow-up was reached by the article by Majmundar et al., on the absolute lymphocyte count as a predictor of mortality and readmission in heart failure hospitalization [16] (Table 4).

**Table 4:** Top performing articles.

**(a) Mendeley reader count**						
Title	Intervention	Editorial	Altmetric Attention Score	Number of citations	**Mendeley reader count**	Tweets
Zhao et al. *Virtual multidisciplinary care for heart failure patients with cardiac resynchronization therapy devices during the Coronavirus Disease 2019 pandemic*[15]	No	Yes	10	3	**71**	5
Bhatia et al. *Subclinical left ventricular dysfunction in COVID-19*[19]	No	No	14	9	**59**	9
Pendell Meyers et al. *Accuracy of OMI ECG findings versus STEMI criteria for diagnosis of acute coronary occlusion myocardial infarction*[14]	Yes	No	185*	16*	**50**	323

**b. Altmetric Attention Score**						
Title	Intervention	Editorial	**Altmetric Attention Score**	Number of citations	Mendeley reader count	Tweets
Pendell Meyers et al. *Accuracy of OMI ECG findings versus STEMI criteria for diagnosis of acute coronary occlusion myocardial infarction*[14]	Yes	No	**185**	16*	50*	323
Sharifkazemi et al. *Three simple but interesting transthoracic echocardiographic road maps for proximal superior vena cava visualisation in healthy young adults*.[17]	No	No	**145**	0	4	230
Guimarães et al. *High risk coronavirus disease 2019: The primary results of the CoronaHeart multi-center cohort study*[18]	Yes	No	**64**	1	43	18

**c. Number of citations**						
Title	Intervention	Editorial	Altmetric Attention Score	**Number of citations**	Mendeley reader count	Tweets
Majmundar et al. *Absolute lymphocyte count as a predictor of mortality and readmission in heart failure hospitalization*[16]	No	No	7	**27**	13	10
Pendell Meyers et al. *Accuracy of OMI ECG findings versus STEMI criteria for diagnosis of acute coronary occlusion myocardial infarction*[14]	Yes	No	185*	**16**	50*	323
Tilz et al. *Very high-power short-duration temperature-controlled ablation versus conventional power-controlled ablation for pulmonary vein isolation: The fast and furious - AF study* [20]	Yes (excluded after randomization)	Yes (Correspondence)	14	**15**	13	27

Top performing articles in a. Mendeley reader count, b. Altmetric Attention Score and c. Number of citations. Asterix indicates that an article also was in top 3 of this category. All articles (n = 186) from 7 subsequent issues (33–39) were included, also articles with editorial.

## 4 Discussion

Herein, we present the results of the #TweetTheJournal study after follow-up of one year after last inclusion. The present social media strategy did not result in statistically significant differences in Mendeley reader counts or citation rate in the intervention group compared to a control group with no dedicated social media strategy. These findings are in line with a previously published study by Tonia et al. [11], in which a social media exposure did not result in significant differences in article downloads and citations. However, our social media strategy resulted in significantly higher Altmetric Attention Score of the articles that received social media promotion through the official journal Twitter/X handle, as compared to those that did not. The upper-bound of followers – representing Twitter/X followers of tweeting accounts and therefore the potential reach on Twitter/X – was significantly higher in the intervention group, compared to the control group. However, this also indicates that social media activity within the control group, which was independent of the intervention, may have potentially influenced the results of the study. In the overall group, we noted a small, but statistically significant correlation of Altmetric Attention Score with Mendeley readers. However, no statistically significant correlations were found between Altmetric Attention Score and number of citations after approximately one year.

Within a large, randomized ESC Journal study, articles published in journals of the ESC family (excluding guidelines or articles providing other forms of recommendations) were selected for randomization according to their potential interest and relevance for readers [9]. If randomized to the intervention group, the tweet included a free link to the full-text version of the article. Articles in the control group were made available according to their publication model, i.e with closed, open or free access. Twitter promotion was associated with a significantly higher citation rate and Altmetric Attention Score and number of tweeting users were significant predictors of numbers of citations [9]. Compared to this study [9], where articles were pre-selected according to their potential interest as judged by the journal’s editors, we included consecutive articles of the same journal and all of them were open-access, not only those within the Twitter/X intervention group. Within the ESC journal family trial, the majority of articles were published in the top 25 % (Q1 – First Quartile) journals of the journal impact factor, which might intrinsically increase the citation and attention potential of each journal. Furthermore, the potential reach of the Twitter/X handle of a well-established journal family as the ESC journal (@ESC_Journal) with over 90 K followers (https://twitter.com/ESC_Journals, access 27.11.2023) is higher than the official IJC H&V Twitter/X handle (@IJC_Heart_Vasc) with 416 followers (https://twitter.com/IJC_Heart_Vasc, access 27.11.2023).

### 4.1 Altmetric Attention Score to measure scientific dissemination

The Altmetric Attention Score (sourced from Altmetric.com) represents a weighted approximation of the online attention gained by a research output, for example for an original article. Different sources are tracked to represent online activity and dissemination and include social media (Twitter/X, LinkedIn, Google + etc.) multimedia platforms, Wikipedia, news, policy documents, Syllabi, patents, and blog posts. It is further based on the volume of people mentioning the source, the source itself (for example news are weighted higher than a tweet) and who the authors are. The latter means that if a professional (researcher, doctor) creates an output, this is weighted higher than an output created automatically by a journal [21]. Mendeley reader counts and citations are not included in the score.

The Altmetric Attention Score therefore measures the impact of online dissemination, whereas traditional metrics as citation rate measures scholarly impact of a research output. In our study, the Altmetric Attention Score was a positive predictor of Mendeley readers in the overall group and in both groups separately. Although the increased Altmetric Attention Score by the intervention did not translate into significantly increased numbers of traditional metrics as number of citations, there were significant correlations of the Altmetric Attention Score with alternative citation indicators, which, in turn, correlated with the number of citations. Nowadays the Altmetric Attention Score is recognized as impact measurement by some academic agencies and grant funding institutions [7]. It is, however, a dynamic score and can fluctuate over time, based on whether an author deletes a source or not. Furthermore, the Altmetric Attention Score can be positively or negatively connotated, as also highly negatively criticized research output generates high attention.

### 4.2 Future implications for social media-based dissemination strategies

Within our main analysis, only articles without editorials were included. In the qualitative analysis, articles with editorials were included to assess the top performing articles of the subsequent issues. The top performing article regarding Mendeley reader count was an article, which received an editorial. Considering the positive results from Ladeiras-Lopez et al. [9], in which articles were selected for randomization according to relevance and potential interest for professionals in cardiovascular medicine, a refined social media strategy including pre-selection, based on the selection for editorials, could be a promising dissemination strategy. Whether a refined Twitter/X promotion strategy with prolonged tweeting period per article may result in increased number of citations in the Twitter/X promotion group should be addressed in subsequent studies. However, a proper control for trial independent social media dissemination, which might bias the impact of the intervention, remains a big challenge.

## 5 Limitations

Our study has several limitations that need consideration. First, the presented results are based on a follow-up period of approximately one year only. Data was, however, retrieved at two points in time and therefore exact follow-up time can vary between articles. Time between publication and data collection was therefore included as offset variable in the negative binomial regression analysis. Also, articles were not stratified based on type of research or whether or not they were related to Covid-19. There was, however, no statistically significant difference of characteristics between both groups. In addition, trial independent social media dissemination and Twitter/X activities occurred. This could have caused crossover bias, which could lead to a dilution of the observed effects of SoMe promotion and therefore an underestimation of the true difference between the intervention and control group.

Finally, the Altmetric Attention Score is a weighted count of attention gained by a scientific paper, with precise methodology of its algorithm being not disclosed.

## 6 Conclusion

Social media promotion of articles did not lead to statistically significant differences the primary endpoint Mendeley reader counts and the secondary endpoint number of citations after approximately one year of follow-up after the last inclusion. Our dedicated social media approach resulted in a higher Altmetric Attention Score, indicating successful and promising impact on online visibility of articles featured through the official journal Twitter/X handle. A longer period of follow-up and refined social media strategy with pre-selection of articles with potential interest to the scientific community should be assessed in subsequent studies.

## CRediT authorship contribution statement

**Konstanze Betz:** Data curation, Writing – original draft. **Melania Giordano:** Writing – review & editing. **Henrike Aenne Katrin Hillmann:** Conceptualization, Data curation, Formal analysis, Writing – original draft. **David Duncker:** Investigation. **Dobromir Dobrev:** Writing – review & editing. **Dominik Linz:** Conceptualization, Data curation, Formal analysis, Investigation, Writing – original draft, Writing – review & editing.

## Declaration of competing interest

The authors declare the following financial interests/personal relationships which may be considered as potential competing interests: Henrike Hillman is Lecture honorary from AstraZeneca and a Fellowship grant from Boston Scientific and Social Media Editor of IJC Heart & Vasculature. Melania Giordano is Managing Editor of IJC Heart & Vasculature. Dobromir Dobrev is Editor in Chief of IJC Heart & Vasculature. Dominik Linz is Associate Editor and Social Media Editor of IJC Heart & Vasculature. Konstanze Betz is Social Media Editor for IJC Heart & Vasculature.
